# Values associated with public involvement in health and social care research: a narrative review

**DOI:** 10.1111/hex.12158

**Published:** 2013-12-10

**Authors:** Felix Gradinger, Nicky Britten, Katrina Wyatt, Katherine Froggatt, Andy Gibson, Ann Jacoby, Fiona Lobban, Debbie Mayes, Dee Snape, Tim Rawcliffe, Jennie Popay

**Affiliations:** ^1^ Mood Disorders CentrePsychologyUniversity of ExeterExeterUK; ^2^ Institute of Health ResearchUniversity of Exeter Medical SchoolExeterUK; ^3^ Division of Health ResearchFaculty of Health and MedicineLancaster UniversityLancasterUK; ^4^ Public Health and PolicyInstitute of Psychology, Health & SocietyUniversity of LiverpoolLiverpoolUK; ^5^ Lancashire Care NHS Foundation TrustMental Health Research NetworkNorth West HubPrestonUK

**Keywords:** engagement, impact, involvement, participation, values

## Abstract

**Background:**

Much has been written about public involvement (PI) in health and social care research, but underpinning values are rarely made explicit despite the potential for these to have significant influence on the practice and assessment of PI.

**Objective:**

The narrative review reported here is part of a larger MRC‐funded study which is producing a framework and related guidance on assessing the impact of PI in health and social care research. The review aimed to identify and characterize the range of values associated with PI that are central elements of the framework.

**Methods:**

We undertook a review and narrative synthesis of diverse literatures of PI in health and social care research, including twenty existing reviews and twenty‐four chapters in sixteen textbooks.

**Results:**

Three overarching value systems were identified, each containing five value clusters. (i) A system concerned with ethical and/or political issues including value clusters associated with empowerment; change/action; accountability/transparency; rights; and ethics (normative values). (ii). A system concerned with the consequences of public involvement in research including value clusters associated with effectiveness; quality/relevance; validity/reliability; representativeness/objectivity/generalizability; and evidence (substantive values). (iii) A system concerned with the conduct of public involvement in including value clusters associated with Partnership/equality; respect/trust; openness and honesty; independence; and clarity (process values).

**Conclusion:**

Our review identified three systems associated with PI in health and social care research focused on normative, substantive and process values. The findings suggest that research teams should consider and make explicit the values they attach to PI in research and discuss ways in which potential tensions may be managed in order to maximize the benefits of PI for researchers, lay experts and the research.

## Introduction

### Definition of public involvement (PI)

We have adopted the following definition of public involvement: the conduct of ‘research carried out **“with”** or **“by”** members of the public rather than **“to**,” **“about**” or **“for”** them’.^1^ Although we recognize there is no consensus, in this paper we have combined ‘involvement’ with the generic term ‘public’, to denote the range of people potentially involved as collaborators in research. This could comprise particular population groups such as older or young people or carers, as well as patients and/or service users.

### Background

Arguments in support of the involvement of the public in health and social care research have been characterized as normative or substantive.^2^ Normative arguments reflect ethical and/or political concerns. They consider involvement as an end in itself, related to values such as rights, justice, fairness and democracy.^3^ In the UK, the public funding of the National Health Service and much research raises questions of public accountability.^4^ Public involvement is also often justified normatively as a route to empower individuals or groups.^5^, ^6^

Substantive arguments focus on the consequences of public involvement. Here, involvement is presented as a means to an end, such as the quality, validity, relevance and/or utility of research. These arguments emphasize the contribution public involvement may make to research including prioritizing different research questions and outcomes; increasing recruitment and improving retention by ensuring research processes are accessible; and assisting with recruitment of participants, data collection, data analysis and dissemination.^7^, ^8^

These arguments reflect the complex historical development of popular movements for greater involvement in research and wider decision making. For example, the disability movement in the late nineteen seventies and early nineteen eighties emphasized ‘rights’, modelling itself on the feminist movement and the black civil rights movement.^9^ In contrast, the mental health involvement movement has a different history and emphasized service users' status as ‘experts by lived experience’.^10^ More recently, the debate has been framed in consumerist terms.^11^ This plethora of values is further elaborated in principles of best PI practice set out in various guidelines in countries such as the USA,^12^ Canada,^13^ Australia^14^ and the UK.^15^ These principles typically highlight ethical values (such as respect for the diversity, rights and autonomy of the public involved)^16^, ^17^, ^18^, ^19^, ^20^, ^21^, the clarity and transparency of involvement processes, and the general accessibility and flexibility of research designs.^22^, ^23^, ^24^, ^25^, ^26^, ^27^

To our knowledge, there has been no previous research explicitly exploring the values about PI held by those involved in health and social care research. However, a survey by the UK Social Policy Association asking 250 members to rank research quality criteria gives an indication of the values held by researchers in this field. Values‐related quality indicators such as transparency (87.8%), patient safety (66.1%), ethical standards (57.8%) and objectivity (43.7%) were rated very highly. In contrast, 35.7% of respondents felt that it was ‘very important’ that service users are consulted about research aims and objectives, 24.9% felt that it was ‘very important that service users were involved appropriately in all stages of research and 21.4% felt that it was ‘very important’ that research has the potential to empower service users.^28^

Although previous writers have identified different value systems associated with public involvement in health and social care research,^29^, ^30^, ^31^ the values operating in this field have not been comprehensively mapped. Such a mapping is an important prerequisite for the development of a better understanding of the impacts of public involvement in health and social care research and the factors shaping these impacts. As the SPA study described above suggests, the values researchers and members of the public hold in relation to PI in research are likely to affect the involvement approaches adopted and hence the kind of impacts this involvement is likely to have.

### Defining values and norms

For the purpose of our review, we adopted the following definition of values: the established collective moral principles and accepted standards of persons or a social group; principles, standards or qualities considered worthwhile or desirable. It was also important to be able to distinguish values from closely related but different concepts particularly that of ‘norms’. Hence, we adopted the following definition of norms as ‘the rule or standard of behaviour shared by members of a social group to which each member is expected to conform’.^32^

Norms are more specific than values and vary depending on both context and frame of reference. For example, while honesty is a value, the ‘rules’ defining honest behaviour in a particular situation are norms and these norms may vary across social groups. The value of ‘clarity’ provides an example of this relationship in the context of PI in health and social care research. We all would probably agree that clarity of communication between researchers and members of the public/service users is an essential principle for successful public involvement in research. A related norm or standard of behaviour often highlighted in guidance on good practice in PI^22^, ^23^, ^24^, ^25^, ^26^, ^27^ is the requirement for terms of reference and/or role descriptions for researchers and public representatives that clearly spell out communication pathways.

### The aim and objectives of the review

The aim of the review reported here was to identify and characterize the range of values associated with PI in health and social care research. The review findings contributed firstly to a Delphi exercise exploring areas of consensus and conflict between different values and secondly to the development of a framework and associated guidance on assessing the impact of PI in research.

The review objectives were to:

1. Search diverse literatures to identify a purposive sample of texts relating to PI in health and social care research.
2. Undertake a thematic analysis of a sample of retrieved texts to develop an initial coding frame for extracting data on values.
3. Extract value statements from a final sample of texts.
4. Conduct a narrative synthesis to identify relationships between the values identified.

### Review methods

Our review focused on existing reviews of empirical research and research methods textbooks. In order to accommodate this diversity, which contained both qualitative and quantitative data, we adopted a narrative approach to synthesis and used a number of analytical tools, including concept mapping^33^.

### Public involvement in the review

The public was involved in the review process in a number of ways. Two service‐user investigators on the research team (DM and TR) contributed to the exploratory searches, development of codes and other group discussions and to the synthesis. The review process and findings were discussed with members of our project public advisory group and our advisory network, who had experience of being involved in health and social care research. Members of the Peninsula Public Involvement Group (PenPIG), a group supported by the NIHR‐Collaboration for Leadership in Applied Health Research and Care in the South West Peninsula,^34^ also helped to write a lay summary and a jargon buster for the review.

### Search strategy

Our challenge was to develop a sampling strategy that allowed for the identification and selection of a diverse yet manageable sample of documents. We employed both comprehensive and purposive sampling methods, focusing on two strands of literature: existing reviews of public involvement in research, and textbooks on health and social care research.

We applied the following inclusion criteria:

Literature from textbooks was included if:

- There was a separate paragraph containing critical analysis or reflection on public involvement in health and social care research.
- There was some reference to at least one of the following: definition, conceptualization, methods, process, measurement, impacts, outcomes of user involvement in health and social care research.
- It was written in the English language, at any time.

Literature for the review of reviews was included if:

- It was a systematic or non‐systematic review.
- It was related to PI in health or social care research (user not subject of research).
- It was written in any language at any time.

As the purpose of the exercise was to extract data about values, irrespective of any methodology used, it was not necessary to conduct critical appraisal of study quality.

We used the following search strategies for the different literatures:

1. A comprehensive review of reviews of empirical research:
  We used the generic and research‐specific part of a search strategy from a Cochrane Review on methods of consumer involvement in developing health‐care policy and research^35^ (see supplementary online material), to screen the Pubmed/MEDLINE, Embase, PsycInfo, ISI Web of Science, ScienceDirect, Wiley, ASSIA and Cochrane databases for reviews of PI in health and social care research. These searches were conducted between February and May 2011 and had no limitation with regards to time periods of publications. We further hand‐searched the INVOLVE, Social Care Institute of Excellence (SCIE) and NIHR Health Technology Assessment libraries (including Mental Health Research Network) as well as the online libraries of several non‐governmental organizations (James Lind Alliance, Joseph Rowntree Foundation, Association of Medical Research Charities, User Involvement in Voluntary Organizations – Shared Learning Group, Folk. Us, TwoCan Associates). Further, elements of the search strategies included reference chaining, hand‐searching key journals (Health Expectations, Health Policy, Int J Cons Studies, Soc Sci&Med, Int J Technol Assess Health Care, J Comm&Appl Soc Psych, Sci Techn & Human Values, Brit J Soc Care, BMJ, Biomed Central Journals), consulting with experts in the field and targeted web searches.

Review of a purposeful sample of textbooks:
  This element of the review included a purposeful sample of textbooks focusing on PI in health and social care research (books, edited books – particularly introductory or overview chapters therein). Library catalogues of two universities were searched with the separate use of the generic search terms of ‘user’, ‘lay’, ‘consumer’, ‘community’, ‘public’, ‘involvement’, ‘engagement’, ‘participation’ and ‘research’ and a sample of relevant textbooks identified.

### Data extraction

Statements or phrases which were consistent with our definition of values were extracted from an initial sample of retrieved literature and grouped thematically. These thematic groups were reviewed and refined in an iterative way with members of the review team. This process resulted in a coding frame and a set of coding rules, which were used to extract data from the full set of included texts (see coding rules and examples of data extraction in supplementary online material).

### Data synthesis

A conceptual mapping approach, as described by Popay *et al*.,^33^ using the mind‐mapping software Inspiration^®^ 9.0 and Microsoft Excel was used for data synthesis. The mapping was used to identify relationships between value statements with elements of shared or common meanings or which co‐occurred. We also applied discrete measures of the quantity of codings (the number of times a certain keyword was mentioned/coded) to our final synthesis.

**Figure 1:**
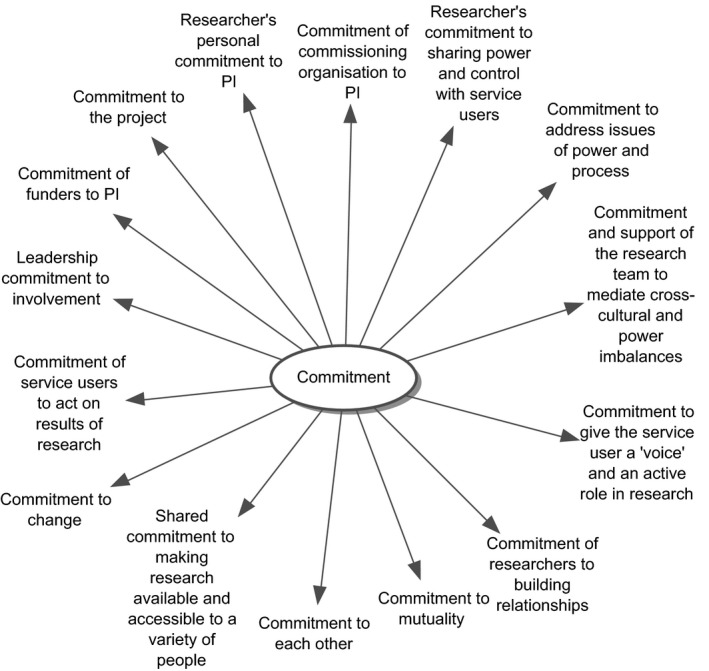
Data synthesis – mapping of value codings on individual value level (grouping all codings related to individual keyword).

**Figure 2:**
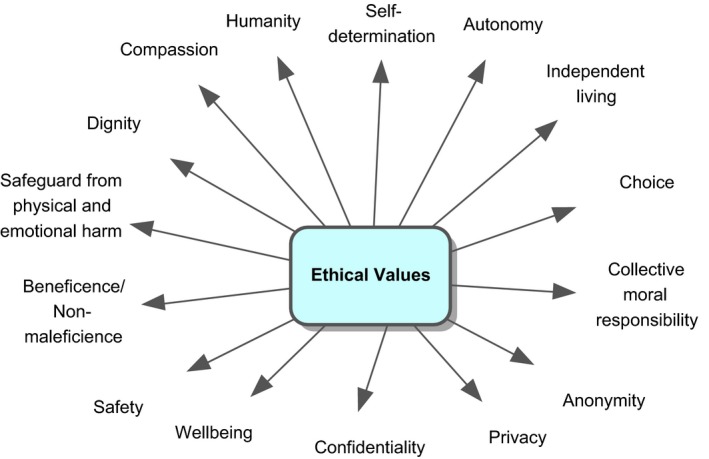
Data synthesis – mapping of value codings on cluster level (as extracted and grouped from statements/paragraphs).

The individual value codings were identified based on our definition of values and were first grouped and clustered around a single value keyword (see example for the value key word ‘commitment’ in Figures 1 and 2). All the codings related to this specific value were then listed and combined into a description or definition based on the various characteristics specified in the statements. A description encapsulating these codes was produced using texts which elaborated the value.

This preliminary synthesis of individual value clusters formed the basis for a final synthesis of value systems – that is, consistent clusters of values – which are described in the Results section below.

### Assessing the robustness of the synthesis

We included critical reflections on the synthesis methods used and the assumptions made at various points in the process by the team of reviewers and the wider project team. All co‐authors contributed to exploratory literature searches and several iterations of data coding exercises to refine our coding frame, coding rules and data synthesis. Additionally, in order to establish the trustworthiness of the synthesis product, we validated our preliminary and final findings with our public advisory group (PAG) on five separate occasions. The PAG feedback and the team's critical reflections led to revisions in the grouping and description of values. We also undertook a Delphi process to further explore differing perspectives on the values identified in the review.

## Results

Forty‐five previous reviews of empirical research were identified and twenty were considered relevant, and were included in the final review of reviews (see supplementary online material). Twenty‐four separate chapters from sixteen textbooks were included (see supplementary online material).

In the 1679 pages of text included in these 44 documents, we coded 1530 value statements.

### Value systems

Our conceptual mapping and thematic coding produced three broad value systems:

1. A value system focused on moral, ethical and/or political concerns associated with PI in research, labelled ‘normative’ values.
2. A value system focused on concerns about the consequences of PI in research, labelled ‘substantive’ values.
3. A value system focused on concerns about the conduct of PI in research, labelled ‘process’ values.

Table 1 shows these three overarching value systems – normative, substantive, process – and the 15 value clusters associated with them.

**Table 1:** Value systems and value clusters

Normative value system: focused on moral, ethical and/or political concerns associated with PI in research	Substantive value system: focused on concerns about the consequences of PI in research	Process value system: focused on concerns about the conduct of PI in research
**Empowerment**: Transfer of control, self‐help, seeking to overcome discrimination and oppression.	**Effectiveness**: PI to actually have an effect on research and implementation.	**Partnership/Equality**: Sharing power and decisions in equal, reciprocal, and collaborative PI processes.
**Rights**: Refers to PI being of intrinsic value, about the fundamental human right to have a say.	**Quality/Relevance**: Increasing the quality, relevance, appropriateness and credibility of research through PI	**Respect/Trust**: Respecting diversity, values, skills, knowledge, and experience in mutually beneficial PI processes.
**Change/Action**: The idea of generating or translating knowledge into action in order to incite change.	**Validity/Reliability**: Processing reliable, valid, and rigorous knowledge through PI.	**Openness/Honesty:** Processes and attitudes being open, honest, flexible, and committed to PI
**Accountability/Transparency**: Public accountability and transparency about research and PI.	**Representativeness/Objectivity/Generalisability:** Creating representative, objective, and generalisable knowledge through PI.	**Independence**: Processes, facilitation, and evaluation being independent.
**Ethical values**: Ethical awareness in order to protect from harm.	**Evidence base**: Generating a substantial, consistent, comparable and replicable evidence base about PI.	**Clarity**: Purpose, processes, communication, and definition of PI being clear.

### Normative value system

This section summarizes the values based on moral, ethical or political elaborations about PI.

#### Empowerment

Normative values were frequently unelaborated (i.e. mentioned in a statement without definition), and understandings of empowerment in particular vary notably.^36^, ^37^, ^38^, ^39^ Empowerment was broadly discussed in the literature as a model or approach that is historically grounded in emancipatory or disability research and informed by a social democratic practice that seeks to overcome discrimination and oppression.^38^, ^40^, ^41^, ^42^, ^43^, ^44^ Some approaches seek to measure this elusive concept through a number of specific variables, such as the number and type of people or communities involved, the number of opportunities for involvement, the degree of involvement in decisions made, or the training or other resources for support that were available.^8^ We came across many competing definitions, and there were recurring personal and essentially political themes that stressed a transfer of control, self‐help, a right to representation and accountability.^38^, ^45^, ^46^, ^47^, ^48^

#### Rights

The literature emphasized the mandate of PI as an intrinsic value per se, as the right to influence publicly funded health and social care research.^4^, ^5^, ^7^, ^8^, ^38^, ^41^, ^46^, ^49^, ^50^, ^51^ Rights were also fundamentally linked to grass‐roots movements like the disability movement, and the civil or welfare rights movement.^40^, ^45^, ^52^, ^53^ They were also centrally embodied as legal or civil rights, political, social and economic rights and responsibilities in the policy agenda of participative democracy, citizenship and consumerism.^38^, ^40^, ^54^, ^55^, ^56^ Finally, these values appeared in professional mandates dedicated to human rights, the right to autonomy and social justice, and securing choice, equal opportunities, welfare and accessibility for service users and carers.^36^, ^38^, ^39^, ^45^, ^53^, ^57^

#### Change/action

Normative value statements related to action and change were most often captured in statements about participatory or action research responding to collective and direct action and/or campaigns for social and political change.^37^, ^38^, ^39^, ^40^, ^46^, ^49^, ^58^ Few of these statements were elaborated beyond a central point about PI seeking to generate knowledge for action or seeking to translate knowledge into action.^7^, ^37^, ^39^, ^45^, ^57^ The kinds of change that were mentioned but not elaborated were social and political change,^40^, ^42^, ^53^ societal and service change,^56^ ‘real’ change,^55^ policy change,^39^, ^47^ transformative change,^54^ user‐led change^45^, effective change,^57^, ^59^ sustainable change,^7^, ^60^ organizational change^37^ and community change.^60^ Mention was also made of changes in the way professionals work^7^ and changed processes of research production.^48^ Elaborated definitions described social change as leading to increased social justice and reduced health inequalities,^43^ and improved health and wellbeing of community members.^39^ These definitions emphasized social change as a form of action.^7^, ^39^

#### Accountability/transparency

PI was often described as a goal in itself, encouraging public accountability and transparency about research.^8^, ^35^, ^37^, ^39^, ^45^, ^46^, ^61^, ^62^ Based on our codings, accountability could be defined as a value that clarifies the relationships between the research and wider society. Public or professional accountability and procedural transparency of the researchers, research team, the general research community, or the project to the research participants, consumer representatives, community members and public was emphasized.^8^, ^36^, ^37^, ^49^, ^51^, ^57^ This value also referred to the general accountability of research funding – especially in a context of transparent public spending, market orientation and managerialism.^7^, ^8^, ^37^, ^38^, ^40^, ^46^, ^51^, ^52^, ^53^, ^57^

#### Ethical values

This value cluster includes established professional codes of ethics that generally encourage the maintenance of an active, personal and disciplinary ethical awareness.^4^, ^8^, ^42^, ^43^, ^44^, ^46^ Further ethical values that emerged from such professional mandates focused on autonomy,^36^, ^37^, ^38^, ^63^ self‐determination and choice, which could be broadly defined as the capacity of individuals and groups to chart their own courses.^37^, ^38^, ^45^, ^56^, ^57^, ^64^, ^65^ The value statements identified further stressed the shared, collective responsibility of researchers to establish processes associated with PI in research that assure the beneficence, wellbeing, humanity and dignity of all those involved.^38^, ^39^, ^43^, ^45^, ^46^, ^57^, ^59^, ^60^ This resonated with other ethical values that stress patient safety (mental and physical) and that seek to protect participants from potential harm as the result of PI in research.^7^, ^40^, ^63^ Confidentiality and privacy were further ethical values that were mentioned in this context.^7^, ^37^, ^61^

### Substantive value system

This section summarizes value clusters that provide statements related to the consequences of PI.

#### Effectiveness

As with some of the value clusters described above (e.g. empowerment), value statements associated with effectiveness covered a range of meanings. One meaning of this term refers to the effectiveness of PI.^8^, ^51^, ^57^, ^63^, ^64^ In the literature, effective PI is presented as leading to increased quality, relevance and impact of research,^4^, ^5^, ^51^ effective dissemination of research findings,^60^, ^65^ appropriate effects on policy and practice,^37^ effective user‐led change,^43^ a more effective health‐care system^46^, ^63^ and better health outcomes.^37^, ^57^

#### Quality/relevance

This value cluster focused on the increased quality of research resulting from PI.^8^, ^35^, ^49^, ^51^ PI is said to improve the quality of research in several ways, for example, by generating research of higher methodological or ethical quality,^37^, ^57^ by increasing the quality of data collected,^8^, ^39^ possibly by using peer interviewers,^7^ by improving the readability and quality of information for research participants^5^ and by providing a better description of the local context, which in turn leads to improved replicability, conceptual robustness and explanatory utility.^39^ This value cluster further resonated with statements about research quality assessment, the evaluation of the quality of involvement^8^, ^39^, ^60^, ^66^ and potential value conflicts in conceptualizing what is considered quality.^8^, ^46^, ^51^

Other dimensions of research quality identified in this value cluster included improvements in the relevance,^4^, ^5^, ^37^, ^47^, ^51^, ^61^ credibility,^7^, ^55^, ^61^ meaningfulness and appropriateness of the research.^7^, ^37^, ^51^, ^67^ In the cases where this was further elaborated, relevance refers to a better, more holistic and responsive focus on patient needs and preferences,^4^, ^7^, ^8^, ^38^, ^39^, ^40^, ^45^, ^46^, ^49^, ^52^, ^59^ – and therefore to the health system as a whole,^56^ asking research questions relevant to the public,^7^, ^8^, ^37^, ^51^ developing research tools which are more meaningful, culturally relevant, sensitive or appropriate to the public,^4^, ^39^, ^51^, ^57^ producing health research of greater clinical relevance^35^ and producing findings that are more relevant to practical decisions made by service users and those caring for them.^37^

#### Validity/reliability

This value cluster is elaborated in discussions of methodological and statistical practices involved in the reliability and validity of assessment tools and measurement instruments.^4^, ^5^, ^8^, ^57^ Specifically in this context, value statements referring to the beneficial impact of PI on the validity and reliability of the developed measures^7^, ^39^, ^55^ and of the collected data and the interpretation of findings were very common.^7^, ^8^, ^46^, ^49^, ^56^, ^68^ Validity was also often used in debates about the nature of knowledge claims.^42^, ^46^, ^50^, ^58^ This included arguments about a hierarchy of evidence and conflicting interest and beliefs about generalisable or positional knowledge.^39^, ^46^, ^69^ With regard to these debates, value statements elaborated what are considered to be traditional scientific research values like neutrality and distance.^40^, ^42^, ^44^, ^53^, ^54^, ^64^, ^69^ In this context, scientific quality, rigour and consistency appeared to be a central feature of the culture of academic, practice and policy communities.^4^, ^5^, ^8^, ^37^, ^44^, ^46^, ^49^, ^50^, ^65^, ^69^

#### Representativeness/objectivity/generalisability

Representativeness arose as a substantive value in the context of statements about population sampling and statistical analysis.^5^, ^37^, ^39^ It further appeared in assessments about the degree to which the study sample was representative of the larger population .^70^ Linked to this, value statements included in this cluster mostly occurred in the context of discussions about the representativeness of the members of the public actually involved in the research process^4^, ^5^, ^40^, ^45^, ^46^, ^54^, ^55^ and how they might be biased,^8^, ^38^, ^43^, ^51^ thus affecting the scientific rigour or objectivity of the study.^4^, ^61^ Frequently, statements questioned whether the public involved were representative of the community being studied because of selectively involving certain people or because of the difficulty in recruiting people from ‘seldom heard groups’.^4^, ^5^, ^8^, ^40^, ^46^, ^65^, ^70^

#### Evidence base for PI in research

This value cluster was elaborated in numerous statements about the need to strengthen the quality of the evidence base about what constitutes best practice in PI, good research management and consistent and robust ways of assessing and reporting the impact of PI on research processes and outcomes.^4^, ^5^, ^7^, ^8^, ^36^, ^46^, ^54^ This was especially elaborated in discussions about the lack of consistency,^7^, ^37^, ^51^, ^57^ comparability^37^, ^57^ and replicability^39^ in quality assessment of PI and its processes and in PI‐related literatures and reporting.^8^ This value was often incorporated into statements about best practice in PI and about criteria for identifying high‐quality, consistent and rigorous research and methods.^7^, ^8^, ^57^ Elaborations of these values also surfaced in normative debates about knowledge, epistemologies and hierarchies of evidence.^37^, ^40^, ^43^, ^44^, ^46^, ^55^ In this context, emphasis was laid on a creative and innovative research environment which was deemed instrumental to the development of a multi‐ or transdisciplinary evidence base.^37^, ^39^, ^45^, ^51^, ^55^, ^57^, ^63^, ^65^, ^68^

### Process value system

The value clusters included in this system mainly arose in elaborations about best practice in PI in research and relate to the processes or the ‘doing’ of involvement.

#### Partnership/equality

Partnership referred to interpersonal relationships between academics, researchers and sometimes health‐care professionals on one hand and service users, consumers and/or community members on the other.^7^, ^38^, ^57^ A key value informing such partnerships was often described as equality.^7^, ^36^, ^37^, ^38^, ^39^, ^40^, ^42^, ^49^, ^52^, ^61^, ^71^ This involves academics and researchers sharing the power they normally hold over the nature of what is researched.^8^, ^38^, ^40^, ^43^, ^45^, ^63^ Equitable partnerships were defined by a gradation of shared responsibility negotiated in collaborative and cooperative decision‐making environments.^7^, ^8^, ^37^, ^39^, ^40^, ^57^, ^60^, ^64^, ^68^ These partnerships, in which equal weight is given to all views,^36^, ^37^ were discussed as being based on principles of mutuality and reciprocity^7^, ^37^, ^47^, ^57^ and a general ethos of reflexivity and learning from each other.^8^, ^37^, ^38^, ^39^, ^44^, ^57^ They were often described to require building and sustaining over time so that all parties understand one another.^7^, ^37^, ^46^

#### Respect/trust

Investing time and work into developing respect and its counterpart trust was described as integral and fundamental to creating and sustaining partnerships between all parties (users, researchers, clinicians, funders and policy makers).^7^, ^8^, ^42^, ^45^, ^46^, ^57^, ^72^ Building trust was typically argued to require time and was described as central to mutually beneficial and lasting relationships, partnerships and collaborations.^8^, ^39^, ^47^, ^57^, ^60^, ^61^ Respect was further described as a working principle essential to successful, sustainable group processes.^7^, ^37^, ^56^, ^57^, ^63^ It also occurred in specific institutional guidance on involvement^16^, ^20^ with regard to respecting the diversity, values, skills, knowledge and experience of public representatives.^8^, ^38^, ^45^, ^54^, ^57^, ^63^, ^64^ In this context, the value of transparency^7^, ^8^, ^35^, ^36^, ^37^, ^40^, ^41^ reflected the importance of building trust for a collaborative enterprise between researchers and service users.^36^, ^41^, ^44^

#### Openness and honesty

When considering the values individuals should bring to PI, the need for an openness of manner^7^, ^8^, ^36^, ^37^, ^38^, ^39^, ^40^, ^41^, ^45^, ^46^, ^49^, ^50^, ^57^, ^63^, ^65^, ^68^, ^72^ and fairness of approach^4^, ^8^, ^44^, ^66^ on all sides was stressed. This would be open and responsive to new ideas, change and advocacy and would foster an environment of flexible decision making.^37^, ^39^, ^45^, ^47^, ^57^, ^63^, ^73^ Further, an attitude of flexibility was often stressed.^37^, ^38^, ^41^, ^45^, ^49^ Based on the statements, this ideally reflects a commitment to involvement and change on the part of all,^7^, ^36^, ^37^, ^41^, ^44^, ^45^, ^50^, ^54^, ^56^, ^57^, ^60^, ^61^, ^70^ as well as a commitment to address issues of power and best practice.^38^, ^47^, ^59^, ^64^ Individual awareness and understanding and a willingness to share opinions and experiences in an honest manner were mentioned as being critical.^7^, ^37^, ^39^, ^41^, ^43^, ^45^, ^50^, ^57^, ^63^, ^64^, ^65^

#### Independence

Independence was mostly elaborated in terms of independent research, that is, seeking to produce evidence which is independent of the particular or potentially conflicting interests of researchers or members of the public involved.^37^, ^38^, ^45^ This was stressed with regard to data collection (e.g. using an external focus group moderator), reviewing and evaluation (e.g. utilizing independent reviewers of outputs, or independent steering committees or an external evaluation of collaborative efforts).^8^, ^37^, ^54^, ^60^ Independence in interactions was also stressed (e.g. members of the public speaking with an independent voice) or through working with independent facilitators (e.g. if a trial design process includes different stakeholder groups seeking consensus).^4^, ^5^, ^8^, ^37^, ^61^, ^72^

#### Clarity

Clarity in the context of the processes of involvement referred to aspects of the coherence of communication,^37^, ^45^, ^63^, ^65^, ^68^ the importance of defining the extent and nature of public involvement and the purpose and agenda of research.^38^, ^59^, ^72^ Value statements highlighted the need for careful expectation management that might use written statements and information sheets, agreements about aims and purposes, as well as role descriptions.^8^, ^37^, ^41^, ^44^, ^46^ Specifically, clarity was mentioned as a guiding value in writing reports about PI, in formulating valid survey questions or defining PI.^5^, ^8^, ^39^, ^50^, ^51^

## Discussion

To our knowledge, this is the first study that explicitly set out empirically to identify and map values associated with PI in health and social care research. This review has produced a new and comprehensive typology of values represented in a broad range of texts about PI in health and social care research. Unlike many of the source documents, this typology explicitly defines the meanings of these values. The development of the typology used an established methodology for synthesizing diverse sources of evidence. Our team represents a wide range of perspectives, but any knowledge production is informed by the perspectives of its producers, and other researchers might have produced a different synthesis.

The following challenges should therefore be considered. Some of the values we identified might not be perceived to be values at all. For example, some values could be regarded as either purposes/aims or impacts/outcomes of involvement, that is, ‘effectiveness’, ‘empowerment’, ‘change’ and ‘action’; expectations and understandings about them may vary. This is further complicated by the finding that values mean different things in different contexts, for example, quality, validity and representativeness.

While this review of reviews and textbooks has identified a wide range of values, it could not associate particular values systems or clusters with particular individuals or different stakeholders (i.e. the question whether public representatives and researchers ascribe to different or overlapping value systems). Furthermore, this cross‐sectional snapshot analysis was not able to identify any trajectories of value formation (the reported impact of changing one's values through involvement^7^ or the possibility that values associated with PI in research have changed over time). It will need a further exploration of these questions in direct exchange with individuals and stakeholders.

This study provides a unique insight into a broad and varied range of value statements that have been synthesized into conceptually robust value clusters and higher‐order value systems. It is an open question whether these values always align with individuals’ actual beliefs, attitudes and behaviour related to PI (i.e. when using the word ‘empowerment’ without elaborating its meaning). There is some indirect evidence about attitudes, mostly however from the perspective of the research community and not the public.^74^, ^75^ One recent study, for example, concluded that health researchers often find themselves torn between political imperatives to involve, strict timelines, a competitive research environment and the necessity of sharing power in research relationships^76^. The values people bring to involvement are likely to have implications for the involvement process and therefore its impacts. Reflecting and clarifying values about involvement before researchers set out to work collaboratively with members of the public could therefore help enhance positive impacts arising from public involvement and avoid negative impacts.

We have used the review findings reported here to develop guidance on how researchers can make explicit their own values‐based rationale for public involvement in research and alert them to the range of values that may be held by members of a research team. This is important because PI can challenge many of the values and assumptions that academic researchers hold. It is very likely that members of research teams will hold different values about PI in research. These differences need to be identified at the beginning of a research project so that strategies for managing potentially conflicting values both within the project team and the wider organizational or funding context can be developed. We suggest that the use of our typology will help make explicit the different values held by individuals in a team, which, if ignored, could lead to tension and disappointment.

We hope that our systematic elaboration of the diversity of values that may be present in such teams will help to improve the practice of public involvement in research.

## Supporting information


**Appendix S1.** Coding Rules.

**Appendix S2.** Happy Values Families – Card Game.

**Table S1.** List of Included/Excluded Systematic Reviews, References, Cochrane Search Strategy, Inclusion Criteria, Jargon Buster

**Table S2.** List of Included Articles from textbooks/edited books
