# Supplementary material for: Values associated with public involvement in health and social care research: a narrative review
Source: Health Expect. 2013 Dec 10;18(5):661–75. doi: 10.1111/hex.12158 (PMC5060838; doi:10.1111/hex.12158)
Supplement: Supplementary file 1 — Appendix S1. Coding Rules. [file HEX-18-0661-s001.doc]

**Supplementary Online Material: Coding Rules**

1. **Coding of text:**

Once a paper has been included into the overall sample (based on inclusion/exclusion criteria) any part of the text is being used for coding. This includes secondary data or findings and direct or indirectly quoted text from other authors or research participants:

| Quote | Extracted meaning unit | Type of Value (Normative, Substantive, Process-related) |
| --- | --- | --- |
| Public involvement is strongly associated with moral and ethical issues, public accountability and transparency encapsulated in the World Health Organisation s declaration of Alma-Ata: ‘the people have the right and duty to participate individually and collectively in the planning and implementation of their health care.’ | right and duty to participate in the planning and implementation of health care | Normative |

1. **Coding of text:**

The quote or value statement has to be implicitly or explicitly related to public involvement in health and social care research:

| Quote | Extracted meaning unit | Type of Value (Normative, Substantive, Process-related) |
| --- | --- | --- |
| ’Questions are raised about who is a service user and the representativeness [scientific value] of service users involved. | representativeness | Substantive |
| As this was a Delphi process, our aim was not to recruit a representative [scientific value] sample, but a diverse panel of experts. | Representative sample | EXCLUDED (No coding) |

1. **Value codings:**

A. Values are only coded if they are explicitly mentioned in the quote (avoid interpretation), B. if not they are successively to be checked for fit to ‘normative debates’ or ‘context factors’:

| Quote | Extracted meaning unit | Type of Value (Normative, Substantive, Process-related) |
| --- | --- | --- |
| Public involvement is strongly associated with moral and ethical issues, public accountability and transparency, encapsulated in the World Health Organisation’s declaration of Alma-Ata: “the people have the right and duty to participate individually and collectively in the planning and implementation of their health care.” | Public accountability | Normative |
| “ | transparency | Normative |
| “ | right and duty to participate | Normative |

1. **Unelaborated Values/Normative debates:**

If a quote or value statement is too general and when Rule 1 cannot be applied, the quote is coded as ‘unelaborated’:

| Quote | Extracted meaning unit | Type of Value (Normative, Substantive, Process-related) |
| --- | --- | --- |
| Public involvement is strongly associated with moral and ethical issues... | Moral and ethical issues | Unelaborated value statement |
| We need to explore what’s good about it and what's bad in different contexts [unelaborated normative debate]. It can't possibly be a wholly positive or negative thing [unelaborated normative debate]. | good vs. bad about UPI in different contexts | Unelaborated normative debate |
| “ | Positive vs. negative | Unelaborated normative debate |

1. **Elaborated Values/Normative debates:**

If a certain value position or normative debate is elaborated more specifically (i.e. through defining the concept, giving examples or listing of characteristics) these are coded separately summarizing the main concept and sub-concepts according to a discerned shift in meaning.

| Quote | Extracted meaning unit | Type of Value (Normative, Substantive, Process-related) |
| --- | --- | --- |
| There may be a moral imperative for public involvement in research in terms of citizenship, accountability, rights etc | moral imperative for public involvement - citizenship | Normative |
| " | moral imperative for public involvement - accountability | Normative |
| " | moral imperative for public involvement - rights | Normative |

1. **Value codings dependent on context:**

Values are coded based on the respective context in which they are uttered. This has to be taken into account and coded respectively making a unique choice where possible (no dual-coding):

| Quote | Extracted meaning unit | Type of Value (Normative, Substantive, Process-related) |
| --- | --- | --- |
| Public involvement is strongly associated with moral and ethical issues, public accountability and transparency... | transparency | Normative |
| The principle of clarity and transparency reflects the importance of building trust for a collaborative enterprise between researchers and service users. | transparency | Process-related |

| Quote | Extracted meaning unit | Type of Value (Normative, Substantive, Process-related) |
| --- | --- | --- |
| Whereas empowerment is very much a political concept related to issues of control and accountability, consumerism stresses economic rights such as satisfaction and value-for-money. | Empowerment - political concept related to issues of control and accountability | Elaborated Normative |
| Involving consumers in research activity may be considered a form of empowerment because it has the potential to strengthen disadvantaged or disempowered groups, and gives individuals the chance to speak out on research issues. | Empowerment - strengthen disadvantaged or disempowered groups | Impact/Outcome (EXCLUDED-NOT CODED) |
| “ | Empowerment - gives individuals the chance to speak out on research issues | Impact/Outcome (EXCLUDED-NOT CODED) |
| However, an empowerment model of research implies that this should be an explicit aim of the research. | empowerment model of research implies that this should be an explicit aim of the research | Model/approach (EXCLUDED-NOT CODED) |

1. **Overlap of normative debates/impacts and values:**

In some cases impacts or normative debates also relate to underlying values – all have to be coded separately:

| Quote | Extracted meaning unit | Type of Value (Normative, Substantive, Process-related) |
| --- | --- | --- |
| However, I think its impact on research is the most important consideration and the fact that it is likely to improve the quality of the research is the strongest argument for advocating it. | improve the quality of the research | Impact/Outcome |
| “ | quality | Substantive |
| ‘...strengthening the evidence base may therefore not only be about finding the most robust and rigorous ways of assessing impact, but also about helping researchers and the public to find the most useful and consistent way of telling their stories | finding the most robust and rigorous ways of assessing impact vs. helping researchers and the public to find the most useful and consistent way of telling their stories | Normative debate |
| “ | robustness | Substantive |
| “ | rigour | Substantive |
| “ | usefulness | Substantive |
| “ | consistency | Substantive |
