# Supplementary material for: Values associated with public involvement in health and social care research: a narrative review
Source: Health Expect. 2013 Dec 10;18(5):661–75. doi: 10.1111/hex.12158 (PMC5060838; doi:10.1111/hex.12158)
Supplement: Supplementary file 2 — Appendix S2. Happy Values Families – Card Game. [file HEX-18-0661-s002.pdf]

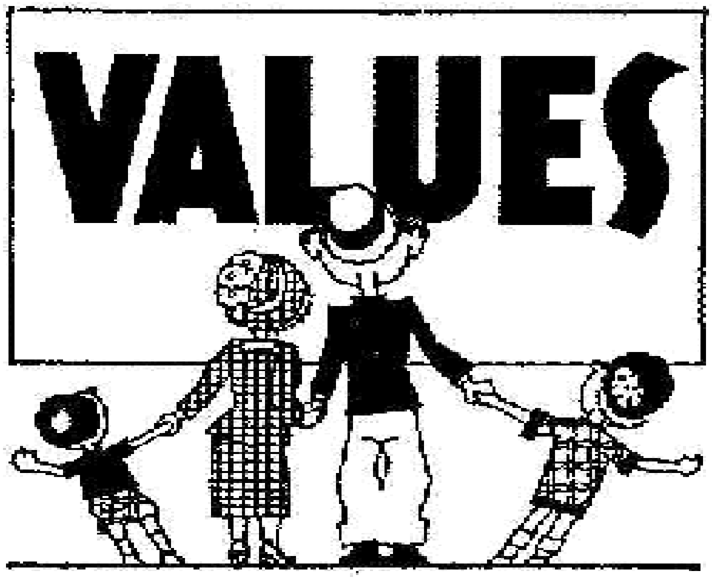

Happy Values Families – Card Game

Values associated with Public Involvement in Health and Social Care Research

Values may inform processes and impacts of PI and hence their assessment. This card game therefore aims to surface and facilitate discussions about what values might be present in any research team engaging with PI in research.

It allows a playful and educative approach to this subject. Its purpose may be to identify the different values that members of the research team hold and/or to generate a set of core values. People can however use the cards in different ways, depending on their own needs, preferences and respective context. As a formal result it might, for example, serve to inform the development of project aims or expected impacts, rules of engagement, role descriptions, or process and impact evaluations..

Instructions:

- Print, cut out and mix the cards (ideally in colour and on A3 paper).
- The aim of the game is to try and collect three cards belonging to one of the three value families.
- There are 15 cards in a pack which allows a maximum team of four members to play at any one time (use two packs for more than five members or play in separate groups of up to four people)
- For any number within the team deal out three cards to each team member and place the leftover cards in the middle (make sure that nobody has three of the same family already!).
- Starting in a circle with the person to the right of the dealer, each player picks up a card from the leftover pack and then discards one card to the leftover pack.
- The winner is the first to put down a set of three cards of the same family.

Please feel free to be creative and make up or add your own rules and play around with the cards in whichever way suits you! Some teams might simply want to give out a whole set of cards to each team member and use the cards in a prioritization exercise (e.g. by collating them in a desired order and based on discussions or majority votes).

The ensuing discussions might also be used to add to or define your own understanding of what certain values or families might mean to you, your team and your project.

Normative Value System: focused on moral, ethical and/or political concerns associated with PI in research

Normative Values Family

Empowerment • Change/Action

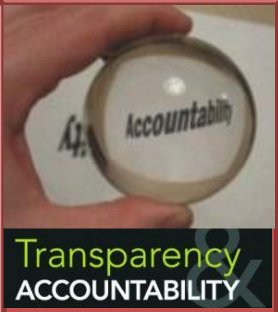

Public accountability transparency about research and PI

Rights • Ethical Values

Accountability/Transparency

Normative Values Family

Empowerment • Change/Action

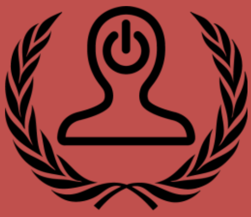

EMPOWERMENT

Transfer of control, self-help, seeking to overcome discrimination and oppression.

Rights • Ethical Values

Accountability/Transparency

Normative Values Family

Empowerment • Change/Action

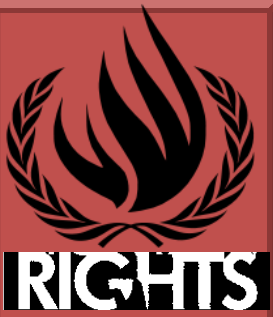

RIGHTS

PI being of intrinsic value, fundamental human right to have a say

Rights • Ethical Values

Accountability/Transparency

Normative Values Family

Empowerment • Change/Action

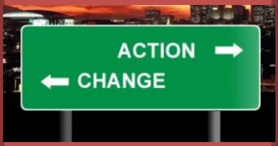

Generating or translating knowledge into action in order to incite change.

Rights • Ethical Values

Accountability/Transparency

Normative Values Family

Empowerment • Change/Action

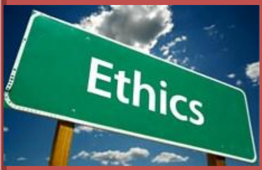

Ethical awareness in order to protect from harm.

Rights • Ethical Values

Accountability/Transparency

Substantive Values Family: focused on concerns about the consequences of PI in research

Substantive Values Family

Effectiveness • Quality/Relevance

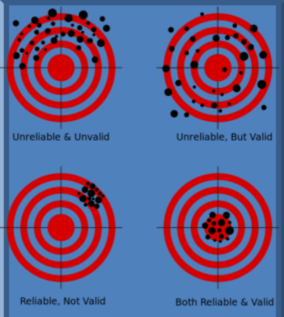

Processing reliable, valid, rigorous knowledge with PI

Representativenesss • Evidence base

Validity/Reliability

Substantive Values Family

Effectiveness • Quality/Relevance

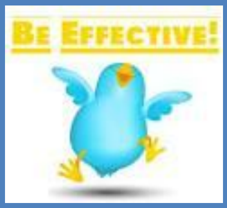

PI to actually have an effect on research and implementation

Representativenesss • Evidence base

Validity/Reliability

Substantive Values Family

Effectiveness • Quality/Relevance

Representative-ness
Objectivity
Generalisability

Creating representative, objective, and generalisable knowledge through PI.

Representativenesss • Evidence base

Validity/Reliability

Substantive Values Family

Effectiveness • Quality/Relevance

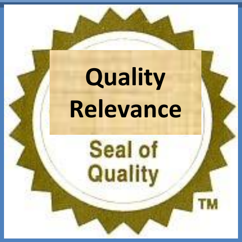

Increasing the quality, relevance, appropriateness and credibility of research through PI

Representativenesss • Evidence base

Validity/Reliability

Substantive Values Family

Effectiveness • Quality/Relevance

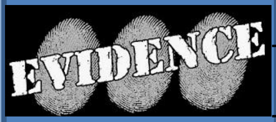

Generating a substantial, consistent, comparable and replicable evidence base about PI.

Representativenesss • Evidence base

Validity/Reliability

Process-related Values Family: focused on concerns about the conduct of PI in research

Process Values Family

Partnership/Equality • Independence

Openness
Honesty

Processes and attitudes being open, honest, flexible, and committed to PI

Openness • Honesty

Respect/Trust • Clarity

Process Values Family

Partnership/Equality • Independence

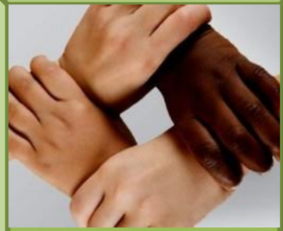

Sharing power and decisions in equal, reciprocal, and collaborative PI processes.

Openness/Honesty/Flexibility/Commitment

Respect/Trust • Clarity

Process Values Family

Partnership/Equality • Independence

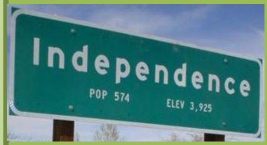

Processes, facilitation, and evaluation being independent.

Openness/Honesty/Flexibility/Commitment

Respect/Trust • Clarity

Process Values Family

Partnership/Equality • Independence

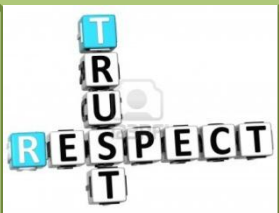

Respecting diversity, values, skills, knowledge, and experience in mutually beneficial PI processes.

Openness/Honesty/Flexibility/Commitment

Respect/Trust • Clarity

Process Values Family

Partnership/Equality • Independence

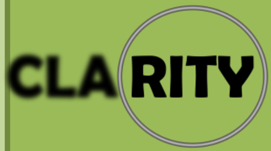

Purpose, processes, communication, and definition of PI being clear.

Openness/Honesty/Flexibility/Commitment

Respect/Trust • Clarity
