# Supplementary material for: Values associated with public involvement in health and social care research: a narrative review
Source: Health Expect. 2013 Dec 10;18(5):661–75. doi: 10.1111/hex.12158 (PMC5060838; doi:10.1111/hex.12158)
Supplement: Supplementary file 3 — Table S1. List of Included/Excluded Systematic Reviews, References, Cochrane Search Strategy, Inclusion Criteria, Jargon Buster [file HEX-18-0661-s003.docx]

Supplementary Online Material; Table 1: List of Included/Excluded Systematic Reviews, References, Cochrane Search Strategy, Inclusion Criteria, Jargon Buster

| **No** | **First Author** | **Year** | **Theme related to User Involvement** | **Lit Range** | **No of docs** | **Origin/Language** | **Pages** | **Comment** | **Source** |
| --- | --- | --- | --- | --- | --- | --- | --- | --- | --- |
| 1 | Allsop([1](#_ENREF_1)) | 2010 | Children, Health Technology Assessment HTA, Methodology | n.a. | n.a. | UK |  | EXCLUDED | MEDLINE Cochrane Search 25.02.2011 |
| 2 | Avard([2](#_ENREF_2)) | 2010 | Health Policy, Health Genomics | 1998-2009 | 70 | Canada – Interntl. |  | EXCLUDED | PsycINFO 09.03.2011 |
| 3 | **Baxter(**[**3**](#_ENREF_3)**)** | 2001 | Health Research, Related Fields | 1995-2001 | 500 | UK – English | **152** | **INCLUDED** | Ref Chaining Smith 2008 02.03.11 |
| 4 | Bee([4](#_ENREF_4)) | 2008 | Mental Health Nursing, User Views | 1994-2005 | 132 | UK only |  | EXCLUDED | Science Direct 17.02.2011 |
| 5 | **Boote(**[**5**](#_ENREF_5)**)** | 2002 | Health Research | n.a. | n.a. | UK | 24 | **INCLUDED** | Ref Chaining Morrow 2010 24.02.11 |
| 6 | **Boote(**[**6**](#_ENREF_6)**)** | 2010 | Health Research, Design Stage | 1995-Jan2009 | 335 | UK - English | 14 | **INCLUDED** | EMBASE 08.02.2011 |
| 7 | **Boote(**[**7**](#_ENREF_7)**)** | 2011 | Public Involvement in Design/Conduct of RCTs | - Nov 2009 | 9 | ? | 26 | **INCLUDED** | Expert advice 18.05.2011 |
| 8 | Braye([8](#_ENREF_8)) | 2005 | Systematic Reviews, Lessons learned | n.a. | n.a. | UK |  | EXCLUDED | Ref chaining Smith Review 21.02.2011 |
| 9 | **Brett(**[**9**](#_ENREF_9)**)** | 2010 | Health and Social Care Research | 1995-End2009 | 83 | UK – English | **292** | **INCLUDED** | MRC Call for funding application |
| 10 | Burton([10](#_ENREF_10)) | 2004 | Community involvement in area-based initiatives | -2003 | 26 + 8 reviews | Uk only |  | EXCLUDED | Ref chaining 31.10.11 |
| 11 | **Cargo(**[**11**](#_ENREF_11)**)** | 2008 | Participatory Research | 1998-2008 | 300 | US –English? | **25** | **INCLUDED** | MEDLINE Cochrane Search 25.02.2011 |
| 12 | **Chen(**[**12**](#_ENREF_12)**)** | 2010 | Community Based Participatory Research CBPR, Dissemination | 2005-End2009 | 101 | US only | 7 | **INCLUDED** | MEDLINE Cochrane Search 25.02.2011 |
| 13 | **Clavering(**[**13**](#_ENREF_13)**)** | 2010 | Health Research, Children | n.a. | n.a. | UK - English | 9 | **INCLUDED** | MEDLINE Search 18.03.2011 |
| 14 | Cook([14](#_ENREF_14)) | 2008 | CBPR, Environmental/Occupational Health | -2007 | 30 | US only |  | EXCLUDED | MEDLINE Cochrane Search 25.02.2011 |
| 15 | Crawford([15](#_ENREF_15)) | 2002 | Health Services, Planning/Development | 1960-Oct2000 | 42 | UK only |  | EXCLUDED | Ref chaining Daykin 2007 09.02.2011 |
| 16 | Daykin([16](#_ENREF_16)) | 2007 | Health Services, Patient Advice Liaison Services | 2000 | 8 | UK only |  | EXCLUDED | EMBASE 08.02.2011 |
| 17 | Dickens([17](#_ENREF_17)) | 2011 | Mental Health, Terminologies, User views | Apr-02 | 11 | UK - English |  | EXCLUDED | Pubmed 24.02.2011 |
| 18 | Doel([18](#_ENREF_18)) | 2007 | Measurement service user and carer participation in social care | 2001-2006 | 48 | English only |  | EXCLUDED | Ref chaining Minogue 2009 30.09.11 |
| 19 | Dowling([19](#_ENREF_19)) | 2004 | Health and Social Care Services | 1997-2004 | 36 | UK only |  | EXCLUDED | Expert advice 17.03.11 |
| 20 | **Fudge(**[**20**](#_ENREF_20)**)** | 2007 | Health Research, Older people | 1995-2005 | 30 | UK – Interntl. | 9 | **INCLUDED** | ASSIA 04.03.2011 |
| 21 | Gagnon([21](#_ENREF_21)) | 2011 | HTA, Research and Process | -2010? | 24 | Canada - Interntl. |  | EXCLUDED | MEDLINE Cochrane Search 25.02.2011 |
| 22 | Gauvin([22](#_ENREF_22)) | 2010 | HTA, Agencies | -March 2008 | 64 | Canada - Interntl. |  | EXCLUDED | Ref chaining Menon 2011 10.03.11 |
| 23 | **Glasby/Clark(**[**23**](#_ENREF_23)**)** | 2004 | Mental Health Services and Research | 1997-2002 | 685 | UK – English? | 8 | **INCLUDED** | Ref chaining Beresford 2006 15.02.11 |
| 24 | **Grant-Pearce(**[**24**](#_ENREF_24)**)** | 1998 | Health research prioritization, professional vs consumer perspective | n.a. | n.a. | UK/Europe | 63 | **INCLUDED** | HTA.ac.uk 04.05.11 |
| 25 | **Hubbard(**[**25**](#_ENREF_25)**)** | 2008 | Cancer Research | 1994-2006 | 52 | UK – Interntl. |  | **INCLUDED** | MEDLINE Cochrane Search 25.02.2011 |
| 26 | Menon([26](#_ENREF_26)) | 2011 | HTA, Coverage/Reimbursement Decisions | n.a. | n.a. | Canada – Interntl. |  | EXCLUDED | MEDLINE Cochrane Search 25.02.2011 |
| 27 | **Minogue(**[**27**](#_ENREF_27)**)** | 2009 | Mental Health Education, Training and Research | 1999-2009 | n.a. | UK only | 17 | **INCLUDED** | SCIE 01.03.2011 |
| 28 | Mitton([28](#_ENREF_28)) | 2009 | Health Services, Priority Setting | 1981-2006 | 301 | UK - English |  | EXCLUDED | MEDLINE Cochrane Search 25.02.2011 |
| 29 | Moore([29](#_ENREF_29)) | 2010 | Health Services, Children/Young people, Decision-Making | 1990-2009 | 25 | UK - English |  | EXCLUDED | PsycINFO 09.03.2011 |
| 30 | **Nilsen(**[**30**](#_ENREF_30)**)** | 2006/10 | Health Policy and Research, Methodologies, Cochrane Revs. | May-09 | 6 | Interntl. | 38 | **INCLUDED** | INVOLVE 23.02.2011 |
| 31 | **Oliver(**[**31**](#_ENREF_31)**)** | 2004 | Research and Development Agenda Setting | -1999 | 286 | UK only | **154** | **INCLUDED** | Ref chaining 02.03.2011 |
| 32 | **Oliver(**[**32**](#_ENREF_32)**)** | 2008 | Health Services Research | n.a. | n.a. | UK only | **13** | **INCLUDED** | MEDLINE 02.02.2011 |
| 33 | Paterson([33](#_ENREF_33)) | 2004 | Complementary Therapies | -2003 | 6 | UK only |  | EXCLUDED | Ref chaining Boote 2010 24.03.2011 |
| 34 | Pivik([34](#_ENREF_34)) | 2004 | HTA, Health Policy, Canada | n.a. | n.a. | Canada – Interntl. |  | EXCLUDED | Ref chaining Brett Review 17.03.2011 |
| 35 | Rose([35](#_ENREF_35)) | 2002 | Mental Health, Change Management | 1987-2002 | 112 | UK only |  | EXCLUDED | Ref chaining Daykin 2007 09.02.2011 |
| 36 | Rummery([36](#_ENREF_36)) | 2009 | Health and Social Care, Partnerships | 1997-2007 | 76 | UK – Interntl. |  | EXCLUDED | Generic OVID Search 17.02.2011 |
| 37 | Simpson([37](#_ENREF_37)) | 2002 | Mental Health Service Delivery | 1966-Oct2001 | 12 | UK - English |  | EXCLUDED | Ref chaining Daykin 2007 09.02.2011 |
| 38 | Smith([38](#_ENREF_38)) | 2007 | Integrated Care Pathways (Cataract, Hip, Knee) | 1993-2003 | 63 | UK only |  | EXCLUDED | ASSIA 04.03.2011 |
| 39 | **Smith(**[**39**](#_ENREF_39)**)** | 2008 | Nursing/Midwifery/Health visiting Research | Jul-04 | 345 | UK | **227** | **INCLUDED** | EMBASE 08.02.2011 |
| 40 | **Staley(**[**40**](#_ENREF_40)**)** | 2009/10 | Health and Social Care Research | 1997-Oct2010 | 129 | UK - English | **137** | **INCLUDED** | MRC Call for funding application |
| 41 | **Stewart(**[**41**](#_ENREF_41)**)** | 2011 | Research Prioritisation, Partnerships | Jan-08 | 258 | UK - English | 10 | **INCLUDED** | Hand Search Health Expectations 02.03.11 |
| 42 | Tenbensel([42](#_ENREF_42)) | 2010 | Health Policy | 2002-2009 | 13 | Interntl. |  | EXCLUDED | MEDLINE Cochrane Search 25.02.2011 |
| 43 | **Venuta(**[**43**](#_ENREF_43)**)** | 2010 | Health Research, Citizen Engagement | n.a. | n.a. | Canada only |  | EXCLUDED | MEDLINE Cochrane Search 25.02.2011 |
| 44 | **Viswanathan(**[**44**](#_ENREF_44)**)** | 2004 | Community Based Participatory Research CBPR | - Mar 2003 | 185 | US/Canada - some int. | 86 | **INCLUDED** | Ref chaining Venuta 2010 14.11.2011 |
| 45 | Weldon([45](#_ENREF_45)) | 2004 | Public Engagement, Genetics | n.a. | n.a. | UK only |  | EXCLUDED | MRC Call for funding application |

Reference List

1. Allsop MJ, Holt RJ, Levesley MC, Bhakta B. The engagement of children with disabilities in health-related technology design processes: identifying methodology. Disability & Rehabilitation Assistive Technology. 2010 Jan;5(1):1-13.

2. Avard D, Jean MS, Gregoire G, Page M. Public involvement in health genomics: The reality behind the policies. International Journal of Consumer Studies. 2010 Sep;34(5):508-24.

3. Baxter L, Thorne L, Mitchell A. Small voices, big noises. Lay involvement in health research: lessons from other fields. Exeter: Washington Singer Press; 2001; Access Date: 310512; [www.invo.org.uk/wp-content/uploads/2012/01/smallvoicesfull2001.pdf](http://www.invo.org.uk/wp-content/uploads/2012/01/smallvoicesfull2001.pdf) - ]. Available from: [www.hfht.org/ConsumersinNHSResearch](http://www.hfht.org/ConsumersinNHSResearch).

4. Bee P, Playle J, Lovell K, Barnes P, Gray R, Keeley P. Service user views and expectations of UK-registered mental health nurses: A systematic review of empirical research. International Journal of Nursing Studies. 2008;45:442-57.

5. Boote J, Telford R, Cooper C. Consumer involvement in health research: a review and research agenda. Health Policy. 2002;61:213-36.

6. Boote J, Baird W, Beecroft C. Public involvement at the design stage of primary health research: A narrative review of case examples. Health Policy. 2010;95:10-23.

7. Boote J, Baird W, Sutton A. Public Involvement in the Design and Conduct of Clinical Trials: A Review. The International Journal of Interdisciplinary Social Sciences. 2011;5(11):91-111.

8. Braye S, Preston-Shoot M. Emerging from out of the shadows? Service user and carer involvement in systematic reviews. Evidence & Policy. 2005;1(2):173-93.

9. Brett J, Staniszewska S. The PIRICOM Study: A systematic review of the conceptualisation, measurement, impact and outcomes of patients and public involvement in health and social care research. London: UK Clinical Research Collaboration (UKCRC); 2010 [12/12/11]; Access Date: 121211; [http://www.ukcrc.org/systematic-review-on-ppi-in-clinical-research/]](http://www.ukcrc.org/systematic-review-on-ppi-in-clinical-research/%5d). Available from: <http://www.ukcrc.org/systematic-review-on-ppi-in-clinical-research/>.

10. Burton P, Goodlad R, Croft J, Abbott J, Hastings A, Macdonald G, et al. What works in community involvement in area-based initiatives? A systematic review of the literature. Home Office Online Report 53/04; 2004; Available from: <http://www.scie-socialcareonline.org.uk/profile.asp?guid=f6fb2d99-9733-40e1-9cce-2dd9061a556e>.

11. Cargo M, Mercer SL. The value and challenges of participatory research: strengthening its practice. Annual Review of Public Health. 2008;29:325-50.

12. Chen PG, Diaz N, Lucas G, Rosenthal MS. Dissemination of results in community-based participatory research. American Journal of Preventive Medicine. 2010 Oct;39(4):372-8.

13. Clavering EK, McLaughlin J. Children's participation in health research: from objects to agents? Child Care Health Dev. 2010 Sep;36(5):603-11.

14. Cook W. Integrating research and action: a systematic review of community-based participatory research to address health disparities in environmental and occupational health in the USA. J Epidemiol Community Health. 2008 Aug;62(8):668-76.

15. Crawford M, Rutter D, Manley C. Systematic review of involving patients in the planning and development of health care. BMJ. 2002;325(7375):1263-4.

16. Daykin N, Evans D, Petsoulas C, Sayers A. Evaluating the impact of patient and public involvement initiatives on UK health services: a systematic review. Evidence & Policy. 2007;3(1):47-65.

17. Dickens G, Picchioni M. A systematic review of the terms used to refer to people who use mental health services: User perspectives. Int J Soc Psychiatry. 2011;21:21.

18. Doel M, Carroll C, Chambers E, Cooke J, Hollows A, Laurie L, et al. Developing measures for effective service user and carer participation. London: Social Care Institute for Excellence; 2007 [updated 30/09/11]; Available from: [www.scie.org.uk/publications/positionpapers/pp09.pdf](http://www.scie.org.uk/publications/positionpapers/pp09.pdf)

19. Dowling B, Powell M, Glendinning C. Conceptualising successful partnerships. Health Soc Care Community. [Review]. 2004 Jul;12(4):309-17.

20. Fudge N, Wolfe CDA, McKevitt C. Involving older people in health research. Age and Ageing. 2007;36(5):492-500.

21. Gagnon M-P, Desmartis M, Lepage-Savary D, Gagnon J, St-Pierre M, Rhainds M, et al. Introducing patients' and the public's perspectives to health technology assessment: A systematic review of international experiences. Int J Technol Assess Health Care. [Research Support, Non-U.S. Gov't]. 2011 Jan;27(1):31-42.

22. Gauvin F-P, Abelson J, Giacomini M, Eyles J, Lavis JN. "It all depends": Conceptualizing public involvement in the context of health technology assessment agencies. Social Science & Medicine. 2010;70(10):1518-26.

23. Clark M, Glasby J, Lester H. Cases for Change: User Involvement in Mental Health Services and Research. Research Policy and Planning. 2004;22(2):31-8.

24. Grant-Pearce C, Miles I, Hills P. Mismatches in Priorities for Health Research between Professionals and Consumers. A Report to the Standing Advisory Group on Consumer Involvement in the NHS R&D Programme. Manchester University: PREST; September 1998 [04.05.2011]; Access Date: 040511; [http://research.mbs.ac.uk/INNOVATION/Portals/0/docs/priorities.pdf]](http://research.mbs.ac.uk/INNOVATION/Portals/0/docs/priorities.pdf%5d). Available from: research.mbs.ac.uk/INNOVATION/Portals/0/docs/priorities.pdf.

25. Hubbard G, Kidd L, Donaghy E. Involving people affected by cancer in research: a review of literature. European Journal of Cancer Care. 2008 May;17(3):233-44.

26. Menon D, Stafinski T. Role of patient and public participation in health technology assessment and coverage decisions. Expert rev. 2011 Feb;11(1):75-89.

27. Minogue V, Holt B, Karban K, Gelsthorpe S, Firth S, Ramsay T. Service User and Carer Involvement in Mental Health Education, Training and Research – A Literature Review. Mental Health and Learning Disabilities Research and Practice. 2009:211-27.

28. Mitton C, Smith N, Peacock S, Evoy B, Abelson J. Public participation in health care priority setting: A scoping review. Health Policy. [Research Support, Non-U.S. Gov't

Review]. 2009 Aug;91(3):219-28.

29. Moore L, Kirk S. A literature review of children's and young people's participation in decisions relating to health care. J Clin Nurs. [Review]. 2010 Aug;19(15-16):2215-25.

30. Nilsen E, Myrhaug H, Johansen M, Oliver S, Oxman A. Methods of consumer involvement in developing healthcare policy and research, clinical practice guidelines and patient information material.: Wiley: Cochrane Database of Systematic Reviews; 2010 [cited 2011]; <http://www.thecochranelibrary.com]>. Available from: <http://www.thecochranelibrary.com>.

31. Oliver S, Clarke-Jones L, Rees R, Milne R, Buchanan P, Gabbay J, et al. Involving consumers in research and development agenda setting for the NHS: developing an evidence-based approach. Health Technol Assess. [Review]. 2004 Apr;8(15):1-148, III-IV.

32. Oliver SR, Rees RW, Clarke-Jones L, Milne R, Oakley AR, Gabbay J, et al. A multidimensional conceptual framework for analysing public involvement in health services research. Health Expectations. 2008;11:72-84.

33. Paterson C. [`]Take small steps to go a long way' consumer involvement in research into complementary and alternative therapies. Complementary Therapies in Nursing and Midwifery. 2004;10(3):150-61.

34. Pivik J, Rode E, Ward C. A consumer involvement model for health technology assessment in Canada. Health Policy. 2004 Aug;69(2):253-68.

35. Rose D, Fleischmann P, Tonkiss F, Campbell P, Wykes T. User and carer involvement in change management in a mental health context: Review of the literature. London: National Co-ordinating Centre for NHS Service Delivery and Organisation Research and Development; 2002.

36. Rummery K. Healthy partnerships, healthy citizens? An international review of partnerships in health and social care and patient/user otucomes. Social Science & Medicine. 2009;69:1797-804.

37. Simpson E, House A. Involving users in the delivery and evaluation of mental health services: systematic review. BMJ. 2002;325(7375):1265-7.

38. Smith E, Ross FM. Service user involvement and integrated care pathways. International Journal of Health Care Quality Assurance. 2007;20(2-3):195-214.

39. Smith E. Service user involvement in nursing, midwifery and health visiting research: A review of evidence and practice. International Journal of Nursing Studies. 2008;45:298-315.

40. Staley K. Exploring Impact: Public involvement in NHS, public health and social care research. Eastleigh: INVOLVE; 2009 [12/12/11]; Access Date: 121211; [http://www.invo.org.uk/posttypepublication/exploring-impact-public-involvement-in-nhs-public-health-and-social-care-research/]](http://www.invo.org.uk/posttypepublication/exploring-impact-public-involvement-in-nhs-public-health-and-social-care-research/%5d). Available from: <http://www.invo.org.uk/posttypepublication/exploring-impact-public-involvement-in-nhs-public-health-and-social-care-research/>.

41. Stewart RJ, Caird J, Oliver K, Oliver S. Patients’ and clinicians’ research priorities. Health Expectations. 2010;Article first published online: 22 DEC 2010:DOI: 10.1111/j.369-7625.2010.00648.x.

42. Tenbensel T. Virtual special issue introduction: Public participation in health policy in high income countries--a review of why, who, what, which, and where? Social Science & Medicine. 2010 Nov;71(9):1537-40.

43. Venuta R, Graham ID. Involving citizens and patients in health research. J Ambulatory Care Manage. 2010 Jul-Sep;33(3):215-22.

44. Viswanathan M AA, Eng E, Gartlehner G, Lohr KN, Griffith D, Rhodes S, Samuel-, Hodge C MS, Lux, L, Webb L, Sutton SF, Swinson T, Jackman A, Whitener L. Community-Based Participatory Research: Assessing the Evidence. Evidence Report/Technology Assessment No. 99. Rockville, MD: Agency for Healthcare Research and Quality; 2004; Available from: [www.ahrq.gov/downloads/pub/evidence/pdf/cbpr/cbpr.pdf](http://www.ahrq.gov/downloads/pub/evidence/pdf/cbpr/cbpr.pdf)

45. Weldon S. Public engagement in genetics: a review of current practice in the UK. Lancaster: Lancaster University Institute for Environment, Philosophy and Public Policy; 2004 [16.03.2011]; Available from: [www.nowgen.org.uk/.../98-Review-of-Public-Engagement-Sue-Weldon-pdf](http://www.nowgen.org.uk/.../98-Review-of-Public-Engagement-Sue-Weldon-pdf).

Generic part of the Search Strategy from the Cochrane Consumers and Communication Review Group specialised register search strategy (Limited to English speaking, reviews):

(“consumer participation” or “patient participation” or “patient advocacy” or “consumer advocacy” or “consumer organisation*” or “consumer organization*”) or (consumer* or stakeholder* or patient* or user* or lay* or client* or disab* or citizen* or communit* or public or advoca* or carer*or caregiver* or parent* or relative*)and (“ social care research” or “health services research” or “peer review research” or “health research” or “research agenda” or “research priorit*” or “research program*”)

Inclusion Criteria:

Inclusions for textbooks part and if reporting the following:

- Some amount of critical analysis or reflection on public involvement in health and social care research (at least separate paragraph on subject)
- Definition/Conceptualisation/Methods/Process/Measurement/Impacts/Outcomes of user involvement in health and social care research
- English language.

Inclusions for review of reviews part and if reporting the following:

- Any Time, Any Language
- Systematic and non-systematic reviews
- Must be related to PI in health or social care research (user not subject of research, e.g. research/resource allocation/rationing decisions/decision-making related to service delivery/development)

**Jargon Buster** (with the help of [www.thefreedictionary.com](http://www.thefreedictionary.com)):

**Values:** the moral principles and beliefs or accepted standards of a person or a social group (e.g. ‘nothing about us without us’).

**Normative Debates:** public discussion that represents at least two different value positions; it should be possible to analyse the different underlying value positions (e.g. generating knowledge vs. inciting political change).

**Systematic Literature Review:** a body of text that aims to review the critical points of current knowledge written on a particular topic.

**Abstract:** a brief summary of a research article typically outlining the completed work’s research focus; the research methods used; the results/findings of the research; and the main conclusions and recommendations.

**Narrative Synthesis**: a form of story-telling that refers to an approach to the systematic review and synthesis of findings from multiple studies that relies primarily on the use of words and text and that offers a set of specific tools and techniques to summarise and explain the findings of the synthesis. <http://www.lancs.ac.uk/shm/research/nssr/research.htm>

**Comprehensive Literature Search:** Central part of a systematic literature review that uses free and subscribed scientific databases and search engines (i.e. <http://scholar.google.com/>) using targeted search strategies (a combination of relevant and overlapping keywords; i.e. ‘values’ and ‘user involvement’) to identify a sample of texts.

**Purposeful Sampling:** a sample is a selection within a population of people or in our case a body of texts. As opposed to ‘random sampling’ where each element has an equal chance of being selected, ‘purposeful’ means that you select elements based on a pre-defined purpose (i.e. in our case identifying the wide variety of value positions).

**Deductive/Inductive approaches:** The difference between induction and deduction is that I came to be certain that the sun will rise each morning because I observed this everyday in my lifetime – which is induction. I could also be certain that the sun will rise again tomorrow, because there is convincing evidence and theory around the movement of the planets and the way the sun itself works – which is deduction.

**Qualitative/Quantitative Methods**: Qualitative research is used to explore and understand people’s beliefs, experiences, attitudes or behaviours. In quantitative research, researchers collect data in the form of numbers.

**Delphi technique:** A forecasting or decision-making technique in which different experts give their professional opinions without knowing what the other experts' opinions are. That is, the experts share their opinions anonymously and discuss them in a series of rounds. These rounds continue until they reach some consensus.
