# Supplementary material for: Values associated with public involvement in health and social care research: a narrative review
Source: Health Expect. 2013 Dec 10;18(5):661–75. doi: 10.1111/hex.12158 (PMC5060838; doi:10.1111/hex.12158)
Supplement: Supplementary file 4 — Table S2. List of Included Articles from textbooks/edited books [file HEX-18-0661-s004.doc]

Supplementary Online Material; Table 2: List of Included Articles from textbooks/edited books:

| **No** | **Reference** | **Type of Reference** | **Theme related to User Involvement** | **Pages** |
| --- | --- | --- | --- | --- |
| 1 | Glasby, Jon (2007) Understanding Health and Social Care. Chapter 7: User Involvement and Citizenship. Bristol: Policy Press, pp. 129-147. | Book chapter in general practice literature | Health and Social Care: User involvement and citizenship | 18 |
| 2 | Fox, Mark / Martin, Peter / Green, Gill (2007) Doing practitioner research. Chapter 8: Service User Involvement. London: SAGE, pp. 130-146. | Book chapter in general research literature | Service User Involvement in practitioner research | 16 |
| 3 | Grant, Gordon / Ramcharan, Paul (2006) Chapter 5: User Involvement in Research in Gerrish, Kate / Lacey, Anne (eds) (2006) The research process in nursing. Oxford: Blackwell Publishing, p. 54-69 . | Book chapter in edited general research literature | User involvement in research | 15 |
| 4.1 | Warren, Janet (2007) Service user and carer participation in social work. Chapter 1: Understanding service user and carer involvement and participation. Exeter: Learning Matters, pp. 1-29. | Book chapter in specific practice literature | Service user and carer participation in social work | 29 |
| 4.2 | Warren, Janet (2007) Service user and carer participation in social work. Chapter 2: Origins and development of service user and carer involvement and participation. Exeter: Learning Matters, pp. 30-45. | Book chapter in specific practice literature | Service user and carer participation in social work | 15 |
| 4.3 | Warren, Janet (2007) Service user and carer participation in social work. Chapter 3: Service user and carer involvement and participation: rhetoric or reality? . Exeter: Learning Matters, pp. 46-61. | Book chapter in specific practice literature | Service user and carer participation in social work | 15 |
| 5.1 | Wallcraft, Jan / Nettle, Mary (2009) Chapter 1: History, Context and Language. In: Wallcraft, J., B. Schrank, et al., Eds. (2009). Handbook of Service User Involvement in Mental Health Research. Chichester, John Wiley & Sons, p. 1-13. | Book chapter in edited specific research literature | Principles and Motives for Service User Involvement in Mental Health Research | 13 |
| 5.2 | Faulkner, Alison (2009) Chapter 2: Principles and Motives. In: Wallcraft, J., B. Schrank, et al., Eds. (2009). Handbook of Service User Involvement in Mental Health Research. Chichester, John Wiley & Sons, p. 13-25. | Book chapter in edited specific research literature | Principles and Motives for Service User Involvement in Mental Health Research | 12 |
| 5.3 | Fulford, Bill / Wallcraft, Jan (2009) Chapter 4: Values. In: Wallcraft, J., B. Schrank, et al., Eds. (2009). Handbook of Service User Involvement in Mental Health Research. Chichester, John Wiley & Sons, p. 37-61. | Book chapter in edited specific research literature | Principles and Motives for Service User Involvement in Mental Health Research | 14 |
| 5.4 | Beresford, Peter (2009) Chapter 13: Control. In: Wallcraft, J., B. Schrank, et al., Eds. (2009). Handbook of Service User Involvement in Mental Health Research. Chichester, John Wiley & Sons, pp. 181-199. | Book chapter in edited specific research literature | Principles and Motives for Service User Involvement in Mental Health Research | 18 |
| 6.1 | McPhail, M / Ager, W (2008) Chapter 1 - Introduction: Good Intentions in A Messy World. In: McPhail, M., Ed. (2007). Service user and carer involvement : beyond good intentions. Edinburgh, Dunedin Academic, p. 1-7. | Book chapter in edited specific practice literature | Service user an carer involvement | 7 |
| 6.2 | Ferguson, Ian (2007) Chapter 7 - Concluding Thoughts: Frustrations and Possibilities. In: McPhail, M., Ed. (2007). Service user and carer involvement : beyond good intentions. Edinburgh, Dunedin Academic,p. 72-77. | Book chapter in edited specific practice literature | Service user an carer involvement | 5 |
| 7 | Hafford-Letchfield, T. (2006). Management and organisations in social work. Chapter 4: Service user involvement and customer care. Exeter, Learning Matters, p. 57-73. | Book chapter in general practice literature | Service user involvement and customer care | 16 |
| 8 | Braye, Suzy (2000) Participation and Involvement in Social Care: An Overview. In: Kemshall, H. and R. Littlechild, Eds. (2000). User involvement and participation in social care : research informing practice. London and Philadelphia, Jessica Kingsley, pp. 9-28. | Book chapter in edited specific practice/research literature | Participation and Involvement in Social Care | 19 |
| 9.1 | Beresford. P, Rose, D. (2009) Chapter 2 - Background. In: Sweeney, A., P. Beresford, et al., Eds. (2009). This is Survivor Research. Ross-on-Wye, PCCS Books, pp. 11-21. | Book chapter in edited specific research literature | Survivor research | 10 |
| 9.2 | Sweeney, A. (2009) Chapter 3 - So what is survivor research? In: Sweeney, A., P. Beresford, et al., Eds. (2009). This is Survivor Research. Ross-on-Wye, PCCS Books, pp. 22-37. | Book chapter in edited specific research literature | Survivor research | 15 |
| 10 | Nolan, M., Hanson, E., Grant, G., Keady, J., Magnusson, L. (2007) 1. Introduction: what counts as knowledge, whose knowledge counts? Towards authentic participatory enquiry. in: Nolan, M., E. Hanson, et al., Eds. (2007). User Participation in Health and Social Care Research. Berkshire, Open University Press, pp. 1-13. | Book chapter in edited specific research literature | User participation in health and social care research | 13 |
| 11 | Entwistle, V (2005) Involving service users in health services research. In: Bowling, A. and E. Shah, Eds. (2005). Handbook of health research methods: investigation, measurement and analysis. Maidenhead, Open University Press, pp. 535-554. | Book chapter in edited general research literature | Involving service users in health services research | 19 |
| 12 | Davis, A (2005) Service User Involvement in Mental Health Research and Development. In: Sallah, D. and M. Clark, Eds. (2005). Research and development in mental health: theory, frameworks and models. Edinburgh, Elsevier Churchill Livingstone, pp. 145-162. | Book chapter in edited general research literature | Service User Involvement in Mental Health Research and Development | 17 |
| 13 | McLaughlin, H. (2009). Service User Research in Health and Social Care. Chapter 1: The development of service user involvement in health and social care research. London, Sage, pp. 1-21. | Book chapter in specific research literature | Service user research in health and social care | 21 |
| 13 | McLaughlin, H. (2009). Service User Research in Health and Social Care. Chapter 2: Why service users bother or why bother involving service users in research? London, Sage, pp. 22-41. | Book chapter in specific research literature | Service user research in health and social care | 19 |
| 14 | Hill S. (2007) Involving the consumer in health research. In: Saks M, Allsop J, editors. Researching health : qualitative, quantitative and mixed methods. London: Sage; 2007, pp. 351-367. | Book chapter in edited general research literature | Consumer involvement in health research | 19 |
| 15 | Peter Beresford (2005) Theory and practice of user involvement in research - Making the connection with public policy and practice in: Lowes, L. and I. Hulatt, Eds. (2005). Involving Service Users in Health and Social Care Research. London, Routledge, pp. 6-17. | Book chapter in edited specific research literature | UI in research | 11 |
| 16 | Sophie Staniszewska, Carole Mockford, Andy Gibson, Sandy Herron-Marx and Rebecca Putz (2011) Chapter 10: Moving forward: understanding the negative experiences and impacts of patient and public involvement in health service planning, development and evaluation. In: Marian Barnes, Phil Cottrell (eds.) (2011) Critical perspectives on user involvement. London: Policy Press, pp. 129-141 | Book chapter in edited specific research/practice literature | Negative impacts of UI | 12 |
